# A de novo CACNA1D missense mutation in a patient with congenital hyperinsulinism, primary hyperaldosteronism and hypotonia

**DOI:** 10.1080/19336950.2020.1761171

**Published:** 2020-05-04

**Authors:** María Carmen De Mingo Alemany, Luis Mifsud Grau, Francisca Moreno Macián, Belén Ferrer Lorente, Sara León Cariñena

**Affiliations:** a Pediatric Endocrinology Unit, Hospital Universitario la Fe, Valencia, Spain; b primary care, Hospital la Plana, Vilareal, Spain

**Keywords:** Congenital hyperinsulinism, CACNA1D, hyperaldosteronism, neurodevelopmental disorder, PASNA, voltage-gated L-type Ca^2+^ channels

## Abstract

Congenital hyperinsulinemic hypoglycemia is the most frequent cause of persistent and recurrent hypoglycemia in the first years of life and in many patients rare genetic variants can be identified. Recently a case of congenital hyperinsulinemic hypoglycemia and a severe neurodevelopmental syndrome due to a mutation in the voltage-gated Cav1.3 Ca^2+^ channel *CACNA1D* gene has been reported which required long-term treatment with diazoxide. This suggested *CACNA1D* variants as a potential cause for this condition.

Here we support this observation by presenting the case of a female child with congential hyperinsulinemic hypoglycemia and primary hyperaldosteronism, aortic insufficiency, pronounced developmental delay, muscle hypotonia, and facial dysmorphias but without seizures. Sequencing of the exome of the child and its parents identified a novel *de novo CACNA1D* missense mutation p.L271 H, replacing a highly conserved residue in a functionally relevant region of the voltage-gated Cav1.3 Ca^2+^ channel. The patient was treated with diazoxide and nifedipine with adequate control of glucose metabolism and blood pressure, and with improvement in muscle tone.

Our findings further confirm the pathogenic role of *CACNA1D* for congentital hyperinsulinemic hypoglycemia and primary aldosteronism. Moreover, we provide evidence that the dihydropyridine Ca^2+^ channel blocker nifedipine, although not considered a first-line treatment for congenital hyperinsulinism, may be beneficial to control blood pressure and neurological symptoms in patients with *CACNA1D* mutations.

## Introduction

Congenital hyperinsulinemic hypoglycemia (HH) is the most frequent cause of persistent and recurrent hypoglycemia in the first years of life. It represents a heterogeneous group of diseases in which there is a dysregulation between plasma glucose concentration and insulin secretion [1–4]. Recent advances in genetics have linked congenital hyperinsulinism with mutations in different genes that play a role in insulin secretion (including ABCC8, KCNJ11, GLUD1, GCK, HADH, SLC16A1, UCP2, HNF4A, HNF1A, HK1, PGM1, PPM2, CACNA1D, FOXA2) [1–4]. Recently also a variant (G403D) in the *CACNA1D* gene has been identified in a subject with diazoxide-dependent congential HH. *CACNA1D* encodes the pore-forming α1 subunit of the voltage-dependent L-type Cav1.3 Ca^2+^ channel [5].

Cav1.3 is one of four members of the so-called L-type Ca^2+^ channels [5,6] which are sensitive to Ca^2+^ channel blockers, including dihydropyridines (DHPs) such as nifedipine and isradipine. Cav1.3 channels are widely expressed in the mammalian organism, including neurons, endocrine cells (endocrine pancreas and aldosterone-producing adrenal cortical cells), sinoatrial node and cochlear inner hair cells [5,6]. Homozygous loss of its pore-forming α1-subunit causes congential deafness and sinoatrial node dysfunction in mice and humans [5,7,8]. In contrast, heterozygous *de novo* variants in humans causing abnormal channel gating permitting enhanced channel activity have been identified as high risk pathogenic mutations leading to autism spectrum disorder with and without intellectual disability. In many patients these variants cause an even more severe neurodevelopmental syndrome mainly reflecting abnormal functioning in neurons and endocrine cells [9–11]. Symptoms described in more severely affected patients include neurodevelopmental delay, intellectual disability, autoaggression, seizures and hypotonia [10–13]. Some patients also present with endocrine symptoms at birth which guide early diagnosis and intervention. So far three cases (variants G403D, I750 M, V259A) with a syndrome presenting with congenitial primary aldosteronism have been described also associated with seizures and neurological abnormalities (PASNA, OMIM # 615474; 10,14). Postnatal transient hypoglycemia with fast recovery upon treatment was also reported in these patients. Interestingly, the *de novo* G403D variant was found in a female with normal aldosterone levels but with congential HH requiring continuous diazoxide treatment for the first five years of life [15]. Other clinical features in this patient resembled PASNA with seizures, severe axial hypotonia, limb spasticity and autism [15]. These observations added *CACNA1D* to the list of potential congential HH genes but reports of additional cases is needed to substantiate this finding.

Here we report a novel de novo *CACNA1D* variant in a second case of a patient with congential HH requiring continuous treatment with diazoxide. In addition this subject exhibited clinical overt primary aldosteronism and therefore represents the first case with both endocrine disorders. Moreover, we also found that the L-type Ca^2+^ channel blocker nifedipine not only controlled high blood pressure but also improved muscle hypotonia. This suggests that nifedipine may be a valuable tool to improve also neurological symptoms in selected patients affected by *CACNA1D* gain-of-function mutations.

## Experimental procedures

To identify a genetic cause for the patient’s condition we performed sequencing using a Custom Focused Exome gene panel (Agilent Technologies) and Illumina technology (NextSeq 500). The coding region and 10 base pairs of flanking intronic sequences of the following gene panel related to hyperinsulinism and syndromic forms of hyperinsulinism was sequenced: ABCC8, GCK, GLUD1, HADH, HK1, HNF1A, HNF4A, INSR, KCNJ11, PGM1, SLC16A1, UCP2, APC2, APPL1, BLK, CDKN1 C, CEL, DIS3L2, H19, HNF1B, HYMAI, IGF1 R, INS, KCNQ1OT1, KDM6A, KLF11, KMT2D, LMNA, MAFA, MPI, NEUROD1, NSD1, PAX4, PDX1, PLAGL1, PMM2, RAP1A, RAP1B and SETD2 genes. In addition we analyzed nuclear genes with mitochondrial involvement, syndromic and non-syndromic neurodevelopmental disorders, that could explain the pathology of the patient (more than 1,200 genes) as well as genes causing Nemaline Myopathy (TPM3, ACTA1, NEB, KLHL41, KLHL40, LMOD3, TPM2, MYPN, CFL2, KBTBD13, TNNT1, MYO18B and RYR3).

Heterozygous variants were filtered by removing non-coding and synonymous variants, and variants in control populations, including the IIS-La Fe Genomic Unit’s own database general population (<0.01) and other databases including the Human Gene Mutation Database, Clin Var Database and the Leiden Open Variation Database.

Copy Number Variation Analysis was performed using the Affymetrix Array-CGH (Array-based Comparative Genomic Hybridization). The results were analyzed using Affymetrix’s Chromosome Analysis Suite software, reviewed by at least two specialists.

The genetic study was conducted in accordance with the Declaration of Helsinki with informed parental consent given on behalf of the child.

## Results

**Figure 1.**
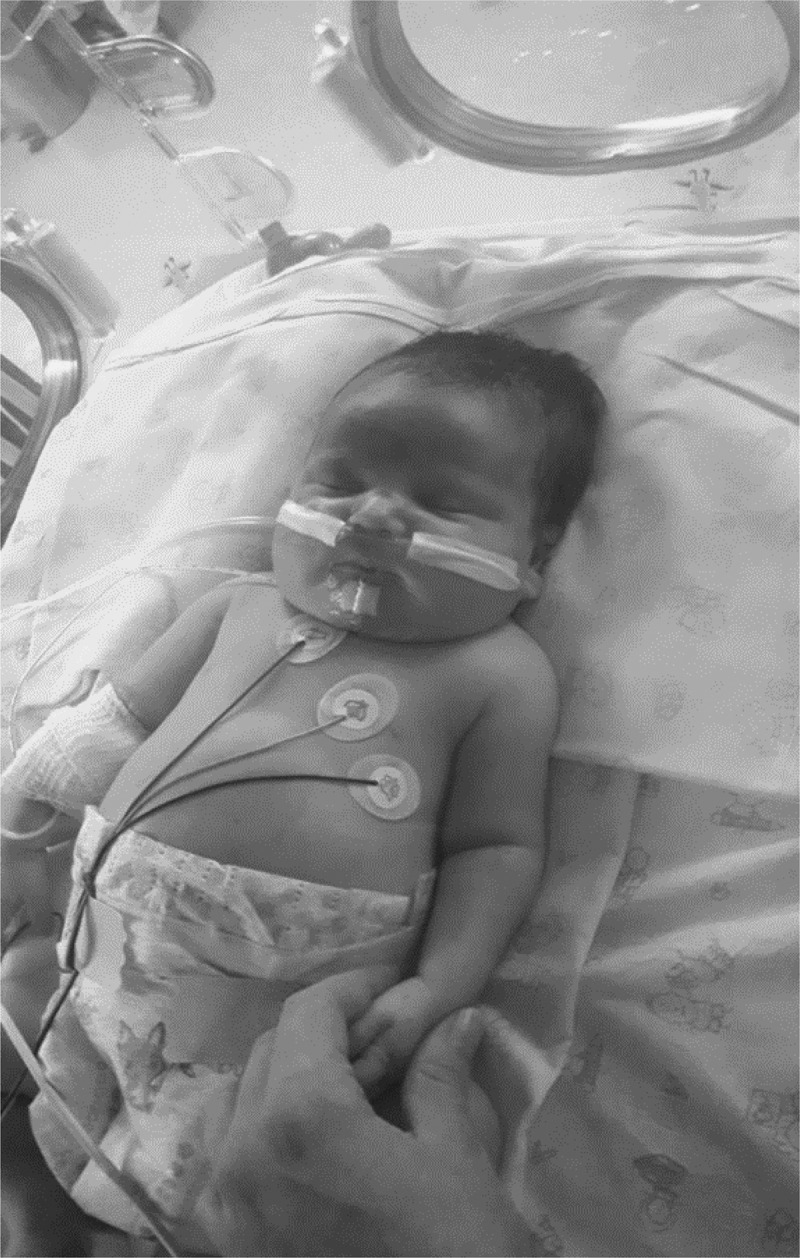
Subject with congenital hyperinsulinemic hypoglycemia. At 48 hours after birth the patients phenotype was characterized by edematous appearance, short neck, low-set ears, a wide nasal bridge and left parietal subgaleal hematoma.

Clinical report: The subject was a newborn female child delivered vaginally at 32 weeks of gestation immediately admitted to the Neonates Unit of the hospital after birth. Premature delivery was due to maternal preeclampsia with HELLP syndrome. The patient had muscle hypotonia, and showed hyporeactivity and tremulations. For this reason capillary blood glucose testing was performed which revealed hypoglycemia at 5 mg/dL. This was treated by an i.v. bolus of glucose followed by continuous glucose infusion. Cord blood gas was pH 7.13, birth weight 2,875 g, Apgar Score 7/8. After birth, she received positive-pressure ventilation with FiO_2_ = 0.21, which was maintained for 2 minutes until spontaneous crying started. She was maintained with PEEP ventilation and transferred to the neonatal ward. Artificial ventilation (BIPAP, CPAP) was continued until spontaneous breathing 6 h after birth. She had an edematous appearance, short neck, low-set ears, a wide nasal bridge and left parietal subgaleal hematoma (Figure 1). There was no family history of hypoglycemia or other syndromic symptoms. There was no consanguinity of the parents.

Because of the tendency for hypoglycemia and in order to try to keep blood glucose above 50 mg/dl), intravenous glucose perfusion was increased up to 12 mg/kg/min. Ketonemia was absent and a glucagon test was compatible with hyperinsulinism: at a blood glucose of 27 mg/dl (reference value 76–110), plasma insulin was 10 microU/ml (3–25), peptide C 2.71 ng/ml (1.10–4.40), cortisol 9.39 µg/dl (6.20–19.40), and growth hormone 13 ng/ml (0.06–5). At 20 hours of life treatment with diazoxide was started (initial dose of 5 mg/kg/day orally at three divided doses every 8 h, followed by 7 mg/kg/day at 72 hours after birth (diazoxide oral suspension of 10 mg/ml which was prepared in the hospital pharmacy). Upon diazoxide therapy blood glucose normalized but she developed intestinal symptoms including vomiting, abdominal distension and absence of stool evacuation without the help of physiological serum enemas. This clinical picture of an intestinal pseudo-obstruction was likely a consequence of a diazoxide-mediated decrease in bowel motility because spontaneous fecal deposition only happened when the dose of diazoxide was decreased. Symptoms persisted until 4 months of life. Abdominal ultrasound, opaque enema and rectal biopsy were normal.

From birth she also exhibited very pronounced axial hypotonia without improvement, trembling and a tendency to lingual protrusion and to maintain closed fists. Therefore an array of tests was performed all with negative results including analysis of blood amino acids, very long chain fatty acids, acylcarnitines, lactic acid, alanine and organic acids in urine). Cerebrospinal fluid study showed a normal profile of neurotransmitters, pterins and folate. In brain MRI punctate hemorrhagic foci were seen in the cerebellum, periatrial areas, hippocampus and semioval centers. At 6 months of age spontaneous stool deposition started without the help of enemas and blood glucose was normal with diazoxide at 5 mg/kg/day. The patient smiled, laughed and babbled, maintained good eye contact with her parents, but still showed pronounced axial hypotonia.

Genetic analysis: The genetic analysis of genes involved in hyperinsulinism, nuclear genes with mitochondrial involvement, syndromic and non-syndromic neurodevelopmental genes, and genes causing Nemaline Myopathy (see Experimental procedures) revealed normal results. No genetic variants were observed that could account for the patient’s pathology.

**Figure 2.**
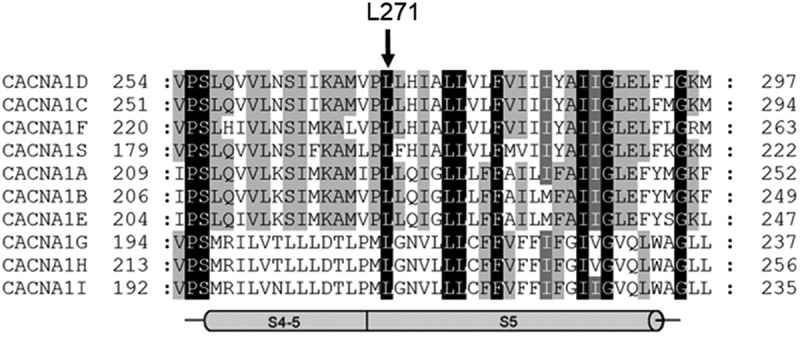
Sequence alignment of all Cav pore forming α1-subunits. L271 is highly conserved in all α1-subunits and located at the cytoplasmic end of the transmembrane helix S5 in repeat I. The following human reference sequences were used for alignment (using Clustal Omega, EMBL-EBI): CACNA1 C: NP_955630.3, CACNA1D: EU363339.1, CACNA1 F: NP_001243718.1, CACNA1 S: NP_000060.2, CACNA1A: NP_075461.2, CACNA1B: NP_000709.1), CACNA1E: NP_001192222.1. CACNA1 G: NP_061496.2, CACNA1 H: NP_066921.2, CACNA1I: NP_066919.2. Conserved identical residues are shown in black shading. The arrow indicates the position of L271 in the Cav1.3 α1 subunit (*CACNA1D* gene). The putative helices S4-S5 and S5 are indicated on the bottom (according to the cryo EM-structure of Cav1.1, 18)

Since severe hypotonia combined with congenital hyperinsulinism had been described in a single subject with a *de novo CACNA1D* missense mutation [15], we performed clinical exome sequencing for genes in which a relationship with neurodevelopmental disorders or some type of neurological involvement at pediatric age had been described. Exome sequencing indeed revealed a potentially pathogenic genetic variant c.812 T > A in the *CACNA1D* gene, which replaces the amino acid leucine by a histidine in position 271 (for reference sequence see legend to Figure 2) of the pore-forming α1-subunit of Cav1.3 L-type Ca^2+^ channels. Conventional Sanger sequencing confirmed the variant in the patient, which was absent in both parents and thus must represent a *de novo* variant. The fact that L271 is conserved in all Cav channels, is located in functionally highly sensitive domain of the channel (cytoplasmic end of the IS5 helix), is absent in the gnomAD database and not present in the parents strongly reinforces the pathological character of this variant.

Since primary aldosteronism was also described in three previous patients carrying pathogenic *CACNA1D de novo* variants [10,14] we also screened the patient for heart disease and hyperaldosteronism. Echocardiography showed a slightly hypertrophic left ventricle with minimal aortic insufficiency. The blood pressure of the patient was elevated (85/53 mmHg). Blood and urine tests were compatible with hyperaldosteronism: serum Na^+^ was 142 mEq/l (reference value 135–150), K^+^ 4.7 mEq/l (3.5–5.5),), aldosterone 88.5 ng/dl (reference values: sitting 7.1/standing 30 ng/dl), renin 16.3 mU/l (sitting 4.2/standing 45.6 mU/l), aldosterone/renin ratio = 5.4 (> 2 is consistent with primary aldosteronism).

At 6 months of life treatment with nifedipine 1 mg/kg/day orally at three divided doses every 8 h (from an oral suspension 5 mg/ml prepared in hospital pharmacy) was added to diazoxide with adequate response on blood pressure. Blood pressure decreased from 100/65 mmHg at 1–3 months to 69-85/42-57 mmHg after the start of nifedipine treatment (months 6–18). Interestingly, this treatment was also associated with a clinically relevant improvement of muscle tone.

At 18 months of life she follow therapy with nifedipine and diazoxide, she is able to bring her hands to the middle line and support her head. She is still unable to sit or crawl. She has oral dyskinesias and continuous movements of the mouth and lingual protrusion. She keeps closed fists and has dystonic hand movements.

## Discussion

This case report adds important clinical information for the diagnosis of individuals affected by congenital HH. First, we report the second patient affected by congenital persistent HH with a pathogenic *CACNA1D* variant who required long-term treatment with diazoxide to control the condition, with adequate clinical response during the first 18 months of life.

Second, identification of *CACNA1D* as the causative gene was important because it guided the further clinical diagnosis of primary aldosteronism and hypertension. Primary aldosteronism was not reported in the first case of congential HH affected by a *CACNA1D* variant G403D [15]. Three individuals with *CACNA1D* variants G403D, I750 M and V259Ahad primary aldosteronism but, despite transient short episodes of postnatal hypoglycemia, did not require long-term diazoxide treatment [10,14]. Therefore our case is the first in a patient with both endocrine disorders, emphasizing the importance of early tests for primary aldosteronism in patients with syndromes affected by *CACNA1D* variants. Like most previously reported *CANCA1D* variants [11–13], L271 also resulted in a severe clinical phenotype not only with endocrine manifestations but also by severe developmental delay, facial dysmorphias and neurological symptoms, in particular muscle hypotonia.

Third, diagnosing a *CACNA1D* variant as the most likely cause for the patient’s conditions also had important therapeutic consequences suggesting a “personalized” approach by treatment with the L-type Ca^2+^ channel blocker nifedipine. There is strong evidence from previous functional studies [9–11] that only heterozygous *de novo CACNA1D* variants inducing gating changes that permit a gain of Cav1.3 channel function are pathogenic. Therefore therapeutic intervention with a nonselective L-type channel blocker appeared reasonable. Since nifedipine is licensed since decades to treat hypertension, we used it to successfully treat the patient’s hypertension (by blocking Cav1.2 L-type channels in arterial smooth muscle [5]. In addition, nifedipine (and some other DHPs) readily permeate the blood-brain barrier and thus may also inhibit excess Cav1.3 channels in the brain [16,17]. Indeed, adding nifedipine to treatment with diazoxide was also associated with a clinically relevant improvement in muscle tone. Since Cav1.3 gain-of function is also responsible for the development of hypoglycemia, this drug that has been classically used for patients with congenital hyperinsulinism, may also contribute to the control of blood glucose.

Taken together our data strongly support *CACNA1D* as a risk gene for congential HH as well as for a severe neurodevelopmental syndrome. Further therapeutic trials are required to confirm our observation that some CNS symptoms, such as muscle hypotonia in our patient, are responsive to treatment with DHP Ca^2+^ channel blockers.
